# Study of JCAD for prognosis and immune infiltration in hepatocellular carcinoma

**DOI:** 10.3389/fimmu.2026.1831629

**Published:** 2026-05-28

**Authors:** Zhonghua Wang, Jing Tang, Siyuan Zhu, Shan Li, Bingyue Yao, Jiayi Tu, Xingyue Zhou, Qinghe Tang, Lan Zhong

**Affiliations:** 1 Department of Gastroenterology, Shanghai East Hospital, Tongji University School of Medicine, Shanghai, China; 2 Department of Hepatobiliary and Pancreatic Surgery, Shanghai East Hospital, Tongji University School of Medicine, Shanghai, China

**Keywords:** biomarker, hepatocellular carcinoma, immune infiltration, JCAD, prognosis

## Abstract

**Background:**

Hepatocellular carcinoma (HCC) remains a common malignant tumor with high morbidity and mortality. Junctional protein associated with coronary artery disease (JCAD), a cell junction-related protein, plays critical roles in multiple pathological processes. However, the prognostic value of JCAD in HCC, particularly its correlation with immune cell infiltration, remains unclear.

**Methods:**

We comprehensively analyzed the biological characteristics of JCAD in HCC using multiple databases and tools. These included The Cancer Genome Atlas (TCGA), Gene Expression Omnibus (GEO), Genotype-Tissue Expression (GTEx), the KM-Plotter platform, and Xiantao Academic tools. Systematic evaluations were performed to assess its protein expression profile, prognostic value, functional enrichment, and immune cell infiltration. Based on TCGA data, we explored correlations between JCAD expression and immune cell infiltration levels. We also assessed whether the impact of JCAD expression on HCC prognosis is partially mediated through immune infiltration. Furthermore, immunohistochemistry assessed JCAD expression in 102 pairs of human HCC and adjacent normal tissues, followed by analysis of its associations with clinicopathological features and disease-free survival (DFS).

**Results:**

Bioinformatics analysis revealed that patients with high JCAD expression had poorer overall survival and showed correlations with gender, tumor stage, and differentiation grade. Notably, JCAD expression was correlated with immune cell infiltration levels, and its association with HCC survival appeared to be partially mediated through immune-related pathways. Clinical validation confirmed JCAD upregulation in 44.1% of HCC tissues. High JCAD expression was significantly associated with portal vein tumor thrombosis and reduced DFS. Kaplan-Meier analysis showed that patients with high JCAD expression had significantly shorter DFS (log-rank p = 0.0012), with lower 1- and 3-year DFS rates compared to the low-expression group. ROC curve analysis indicated that JCAD has modest predictive power for DFS, with an AUC of 0.695 (0.592-0.798).

**Conclusion:**

Elevated JCAD expression may serve as a prognostic biomarker for HCC patients, potentially through immune-related mechanisms. By integrating bioinformatics analysis with clinical validation, this study provides novel evidence supporting the role of JCAD in HCC prognosis and its potential association with immune regulation.

## 1 Introduction

Hepatocellular carcinoma (HCC) ranks as the sixth most prevalent malignancy globally and represents the third leading cause of cancer-related mortality (following lung and colorectal cancers), imposing a substantial societal burden (1, 2). Despite expanding global implementation of surgical and locoregional therapies (3), the 5-year postoperative survival rate remains suboptimal at 40-50%, with 50-60% of HCC patients ultimately requiring systemic therapies (4–6). Recent paradigm shifts in therapeutic strategies have emerged, supported by accumulating clinical evidence demonstrating the efficacy of immune checkpoint inhibitors (ICIs) (7). Chemotherapy-induced tumor cell apoptosis has been shown to potentiate immune activation and to enhance antitumor efficacy. However, despite this potentiation of immune activation, tumor-mediated immune tolerance mechanisms suppress effective immune recognition and cytotoxicity, contributing to disease progression (8, 9).

Junctional protein associated with coronary artery disease (JCAD), an endothelial protein, plays pivotal roles in diverse pathological processes. Current evidence demonstrates that JCAD overexpression in HCC cells promotes tumor proliferation. Moreover, significantly elevated JCAD levels are observed in HCC tissues compared to adjacent non-tumor tissues (10). Experimental studies further indicate that JCAD silencing attenuates hydrogen peroxide (H_2_O_2_)-induced cellular damage, while suppressing apoptosis, inflammatory responses, and vascular endothelial dysfunction (11). Nevertheless, the prognostic relevance of JCAD expression to overall survival (OS) and its association with tumor immune cell infiltration in HCC remain unexplored.

To address these gaps, we combined two strategies: bioinformatic analyses of public datasets and direct experimental validation using clinical samples. We perform immunohistochemical analysis of JCAD expression in a well-characterized cohort of 102 paired HCC and adjacent non-tumor tissues to validate its protein-level dysregulation. Subsequently, we systematically correlate JCAD expression levels with detailed clinicopathological features and patient survival outcomes. Furthermore, this work pioneers the exploration of the relationship between JCAD overexpression and tumor immune infiltration dynamics. This aims to uncover its potential role in the HCC microenvironment and assess its candidacy as a prognostic biomarker and novel therapeutic target.

## 2 Materials and methods

### 2.1 Data collection

The Tumor Immune Estimation Resource (TIMER) web server (https://timer.cistrome.org/) (12, 13) was utilized to reanalyze gene expression data across 32 cancer types and evaluate tumor-infiltrating immune cell levels. The GSE14520 dataset was downloaded to perform survival analyses. The “TIMER-Gene” module was employed to explore correlations between JCAD expression and immune infiltration levels in HCC patients. The “Immune-Gene” module, using multiple algorithms, was applied to analyze associations between JCAD expression and immune infiltration in the TCGA database.

### 2.2 Clinical Samples and immunohistochemical staining

HCC tissue samples were procured from patients undergoing surgical therapy or preoperative puncture at Shanghai East Hospital. Written informed consent was obtained from all participants prior to sample collection. The study was conducted with the approval of the Human Participants Ethics Committee of Shanghai East Hospital ([2023] Research Approval No. 132). Subsequent pathological verification and categorization of all tumor specimens were performed by seasoned clinicians. The main characteristics of the analysis included the following: age, gender, histologic grade, tumor size, tumor numbers, AFP, macrovascular invasion, microvascular invasion, portal vein tumor thrombosis, and DFS. The immunohistochemistry procedure was as follows. First, deparaffinization and rehydration were performed by immersing the tissue sections in xylene for 10 minutes, repeated three times. This was followed by rehydration through a graded ethanol series from high to low concentration. Subsequently, antigen retrieval was conducted by heating a pH 6.0, 0.01 M sodium citrate buffer in a microwave until boiling, then placing the antigen-retrieved slices in the buffer. The slices were washed with PBS three times for 5 minutes each. Next, the slices were incubated with a blocking solution containing normal goat serum at room temperature for 20 minutes. After discarding the blocking solution, an appropriate amount of primary antibody (1:100 dilution) was added, and the slices were incubated overnight at 4 °C. The following day, the slices were warmed at 37 °C for 30 minutes. The primary antibody was then removed, and the slices were washed with PBS three times. Subsequently, a secondary antibody was added and incubated at 37 °C for 1 hour. After incubation, the secondary antibody was discarded, and the slices were washed with PBS three times. DAB staining solution was then applied and developed in the dark at room temperature for 5–10 minutes, wtih staining intensity monitored under a microscope. After staining was completed, the slices were washed with PBS for about 10 minutes to stop the staining. Hematoxylin was used to counterstain the nuclei for 2 minutes, followed by differentiation in hydrochloric acid alcohol solution. The slices were then rinsed with tap water for 10–15 minutes. Finally, dehydration, cleaing, mounting, and microscopy were performed.

### 2.3 Xiantao platform

The Xiantao platform (https://www.xiantao.love/) was used to obtain JCAD expression and prognostic data. A nomogram based on JCAD expression levels and clinicopathological parameters was constructed to predict 1-year, 3-year, and 5-year OS. Calibration curves were generated to assess the nomogram’s predictive accuracy. The “Immune Infiltration” analysis module evaluated relationships between immune cell markers and JCAD expression. Additionally, Gene Set Enrichment Analysis (GSEA) was performed to explore potential mechanisms of JCAD.

### 2.4 GEPIA2

GEPIA2 (https://gepia2.cancer-pku.cn/) (14) was used to obtain data for the top 100 genes correlated with JCAD, focusing on TCGA and GTEx RNA sequencing data. Associations between JCAD and the top three correlated genes were analyzed.

### 2.5 STRING

The STRING database (https://string-db.org/) (15) was used to construct a protein-protein interaction (PPI) network for JCAD. The search parameters were as follows: protein name (“JCAD”), species (“Homo sapiens”), interaction sources (“textmining” and “experiments”), minimum required interaction score (low confidence threshold, score 0.150), maximum number of interactors to show (no more than 50 interactors in the first shell), with other settings as “default”.

### 2.6 Statistical analysis

#### 2.6.1 Analysis of external public database

The prognostic potential of JCAD in hepatocellular carcinoma (HCC) was initially explored using the Kaplan-Meier Plotter database. Within this cohort, patients were stratified into high- and low-expression groups based on the median expression level of JCAD. Differences in overall survival (OS) were assessed accordingly.

#### 2.6.2 Analysis of data from the present study

Statistical analyses were performed using SPSS 22.0 (IBM Corp.) and GraphPad Prism 6.0. Continuous variables were tested for normality using the Shapiro-Wilk test. Normally distributed data are presented as mean ± standard deviation and were compared between two groups using the independent samples t-test. Non-normally distributed data are presented as median (interquartile range) and were compared using the Wilcoxon rank-sum test. Categorical variables are presented as frequency (percentage) and were compared using Pearson’s chi-square test, with Fisher’s exact test applied when any expected frequency was less than 5.

Survival analyses specific to our cohort were conducted to evaluate the prognostic value of JCAD expression. Differences in disease-free survival (DFS) between the high- and low-JCAD expression groups were visualized using the Kaplan-Meier method. These differences were statistically compared with the log-rank test. Univariate Cox proportional hazards regression was also performed. The predictive accuracy of the JCAD gene for 3-year DFS was evaluated using time-dependent receiver operating characteristic (ROC) curve analysis, which accounts for censored survival data, and quantified by the area under the curve (AUC). For all statistical tests, a two-sided p-value < 0.05 was considered statistically significant.

## 3 Results

### 3.1 Analysis of JCAD gene expression in hepatocellular carcinoma

**Figure 1:**
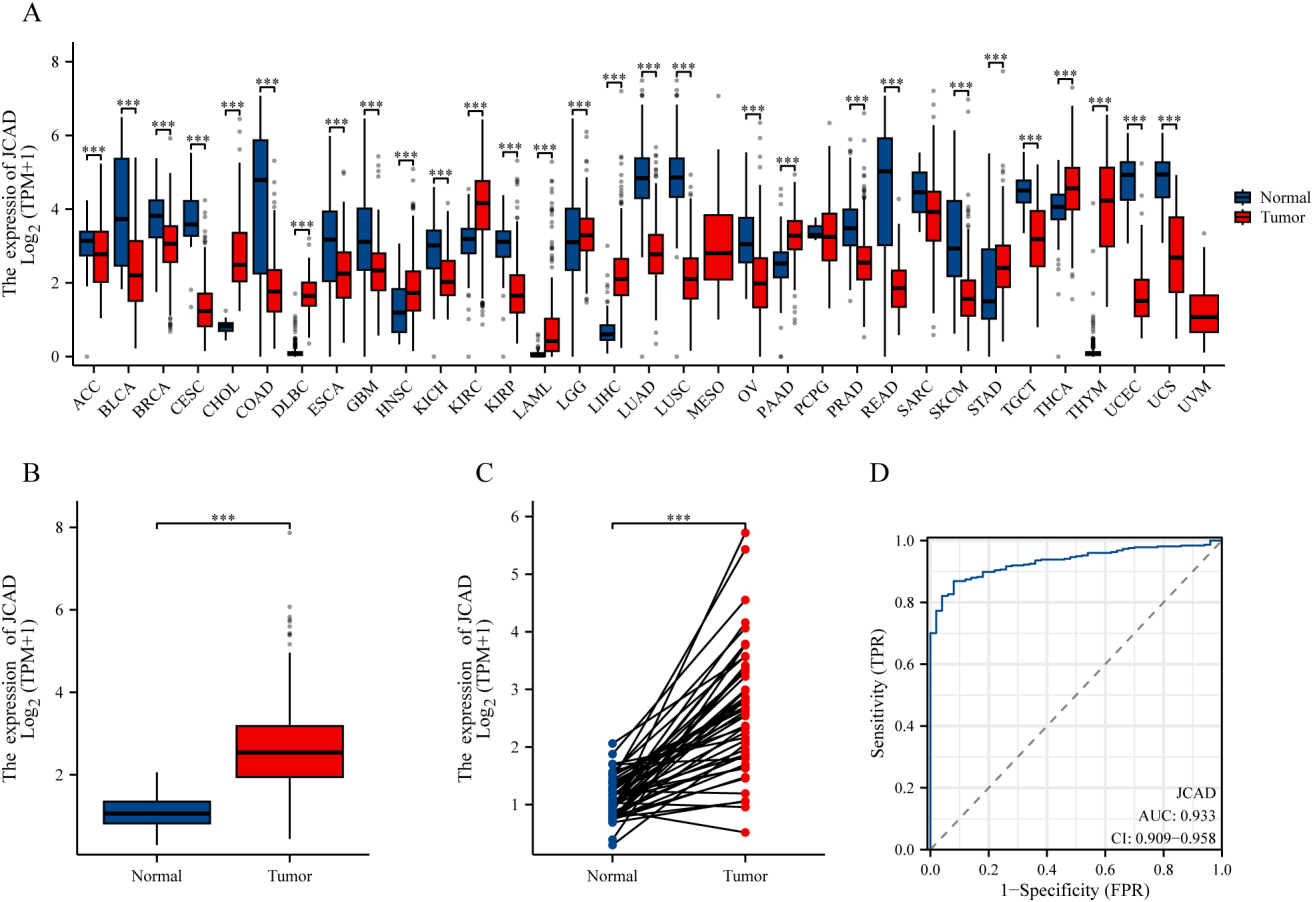
JCAD expression in HCC. **(A)** JCAD expression across multiple cancers analyzed using TCGA and GTEx data. **(B)** The expression level of JCAD was compared between tumor and normal tissues in the TCGA HCC dataset. **(C)** JCAD mRNA expression was analyzed in 50 paired HCC and adjacent normal tissues from TCGA. **(D)** Diagnostic value of JCAD in HCC. p-value significant codes: ***p < 0.001.

Using the Xiantao online tool for pan-cancer analysis of TCGA and GTEx databases across 33 cancer types (**Figure 1A**), CAD mRNA was found to be upregulated in 14 cancer types, including hepatocellular carcinoma (HCC). Compared to normal liver tissues, JCAD expression was significantly elevated in HCC tissues (p < 0.001) (**Figure 1B**). Validation using 50 paired tumor and adjacent normal tissues confirmed marked upregulation of JCAD in tumor samples (p < 0.001) (**Figure 1C**). Addtionally, receiver operating characteristic (ROC) curve analysis demonstrated an area under the curve (AUC) of 0.933 for the prediction of 1-year recurrence (**Figure 1D**), underscoring its diagnostic potential.

### 3.2 JCAD upregulation predicts poor prognosis in hepatocellular carcinoma

**Figure 2:**
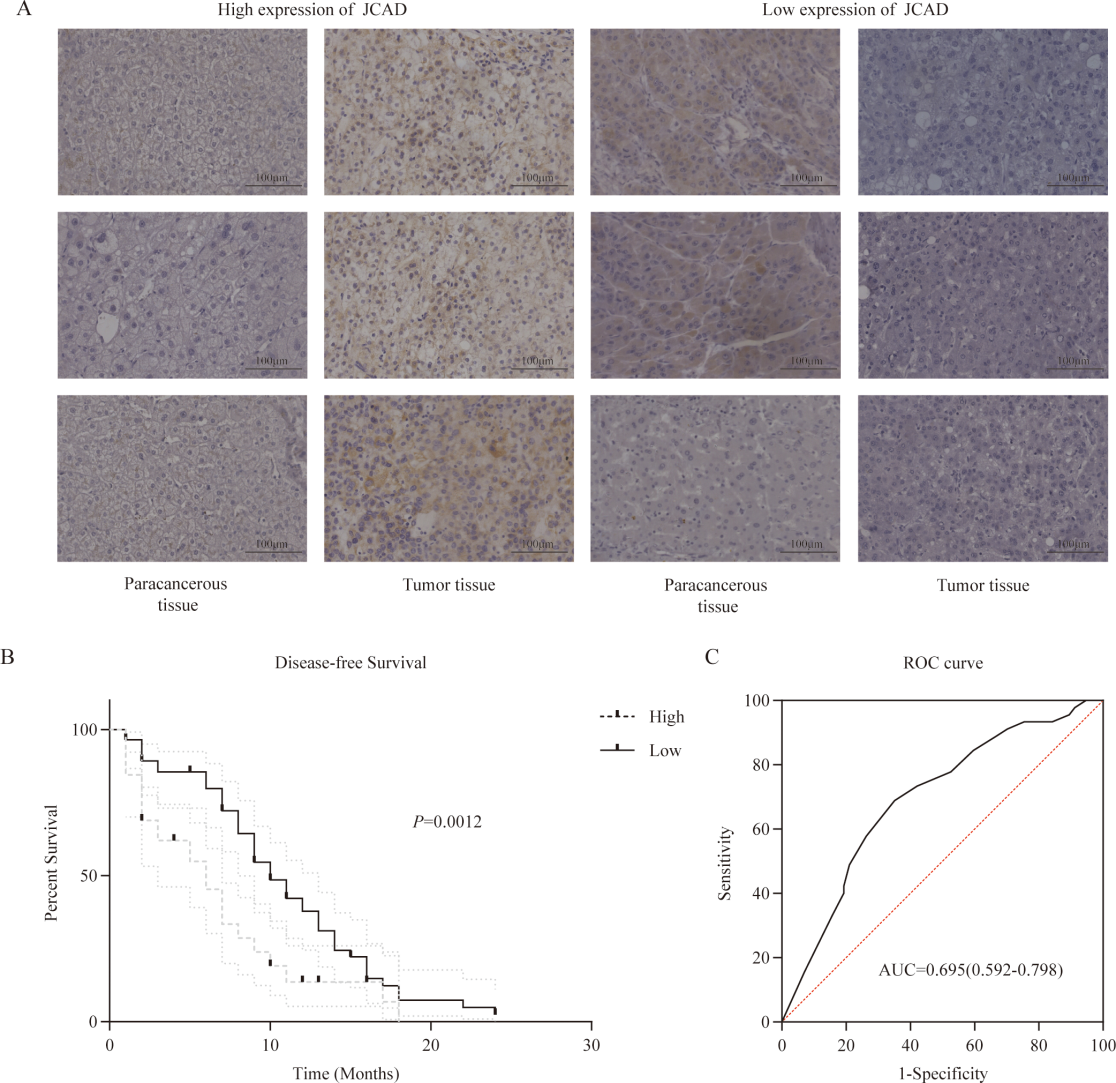
Correlation between JCAD expression and prognosis in hepatocellular carcinoma (HCC) patients. **(A)** Representative immunohistochemical images of JCAD expression in HCC tissues and matched adjacent normal tissues from 3 cases with high expression and 3 cases with low expression (Scale bar: 100 μm). Expression was assessed in 102 paired samples. **(B)** Kaplan-Meier curves comparing disease-free survival (DFS) between HCC patients with high and low JCAD expression. Statistical significance was determined by the log-rank test (p = 0.0012). **(C)** Receiver operating characteristic (ROC) curve evaluating the predictive power of JCAD expression for DFS.

Immunohistochemistry was employed to measure the expression of JCAD in 102 samples of human hepatocellular carcinoma (HCC) tissues and corresponding paracancerous tissues (**Figure 2A**). JCAD was found to be overexpressed in 44.1% of HCC tissues samples compared with corresponding paracancerous tissues (**Table 1**). Based on the clinicopathological features, JCAD overexpression was significantly associated with portal vein tumor thrombus (p = 0.005); and disease‐free survival (DFS) (p < 0.001), but not significantly correlated with age (P = 0.471), gender (p = 0.622), histologic grade (p = 0.180), tumor size (p = 0.412), tumor number (p = 0.530), alpha-fetoprotein (AFP) (p = 0.173), macrovascular invasion (p = 0.318), or microvascular invasion (p = 0.449) (**Table 1**).

**Table 1:** Patient demographics and baseline characteristics.

Characteristic	Overall N = 102^1^	JCAD relative expression		Statistic^2^	P-value
		High N = 45^1^	Low N = 57^1^		
Age	62 (52, 71)	60 (50, 71)	64 (56, 71)	1,175.00	0.471^3^
Gender				0.24	0.622^4^
Female	18 (17.6%)	7 (15.6%)	11 (19.3%)		
Male	84 (82.4%)	38 (84.4%)	46 (80.7%)		
Histologic grade					0.180^5^
G1	6 (5.9%)	1 (2.2%)	5 (8.8%)		
G2	57 (55.9%)	24 (53.3%)	33 (57.9%)		
G3	37 (36.3%)	20 (44.4%)	17 (29.8%)		
G4	2 (2.0%)	0 (0.0%)	2 (3.5%)		
Tumor size				0.67	0.412^4^
<5cm	59 (57.8%)	24 (53.3%)	35 (61.4%)		
≥5cm	43 (42.2%)	21 (46.7%)	22 (38.6%)		
Tumor number					0.530^5^
Multiple	11 (10.8%)	6 (13.3%)	5 (8.8%)		
Single	91 (89.2%)	39 (86.7%)	52 (91.2%)		
AFP (ng/ml)				1.85	0.173^4^
<200	79 (77.5%)	32 (71.1%)	47 (82.5%)		
≥200	23 (22.5%)	13 (28.9%)	10 (17.5%)		
Macrovascular invasion					0.318^5^
No	98 (96.1%)	42 (93.3%)	56 (98.2%)		
Yes	4 (3.9%)	3 (6.7%)	1 (1.8%)		
Microvascular invasion				0.57	0.449^4^
No	89 (87.3%)	38 (84.4%)	51 (89.5%)		
Yes	13 (12.7%)	7 (15.6%)	6 (10.5%)		
Portal vein tumor thrombus				8.00	0.005^4^
No	81 (79.4%)	30 (66.7%)	51 (89.5%)		
Yes	21 (20.6%)	15 (33.3%)	6 (10.5%)		
DFS(month)	7.5 (3.0, 11.0)	6.0 (2.0, 9.0)	9.0 (6.0, 13.0)	783.50	<0.001^3^

1 Median (Q1, Q3); n (%).

2 Wilcoxon rank sum test; Pearson's Chi-squared test; Fisher's exact test.

3 Wilcoxon rank sum test.

4 Pearson's Chi-squared test.

5 Fisher's exact test.

According to the Kaplan-Meier survival curve, HCC patients with high JCAD expression had reduced DFS (**Figure 2B**). Statistical analysis showed that the P-value of the Mantel-Cox test was 0.0012, affirming a significant difference between the high expression and low expression groups. The DFS at 1 year was 37.8% (95% CI 26.4 to 54.0) in the low JCAD expression group and 13.6% (95% CI 6.26 to 29.7) in the high JCAD expression group, respectively. Moreover, the DFS at 3-years was 2.47% (95% CI 0.36 to 16.9) in the low JCAD expression group (**Table 2**). The median DFS was 0.8 years (95% CI 0.8 to 1.1) for the low JCAD expression group and 0.5 years (95% CI 0.3 to 0.7) for the high JCAD expression group (**Table 3**). Furthermore, the time-dependent receiver operating characteristic (ROC) curve was used to assess the predictive power of JCAD for DFS prediction. As shown in **Figure 2C**, the area under the curve (AUC) for DFS was 0.695 (0.592-0.798). These findings indicated that upregulation of the JCAD gene was associated with a poor prognosis in HCC patients.

**Table 2:** Kaplan-Meier estimates for survival rates (95% CI).

Characteristic	N	Event N	1-year	3-year	P-value^1^
Overall	102	86	27.1% (19.3%, 38.0%)	1.66% (0.24%, 11.3%)	
JCAD	102	86			0.0012
Low			37.8% (26.4%, 54.0%)	2.47% (0.36%, 16.9%)	
High			13.6% (6.26%, 29.7%)	0% (—, —)	

**Table 3:** Median survival (95% CI).

Characteristic	Median survival (years)
Overall	0.7 (0.6, 0.8)
JCAD	
Low	0.8 (0.8, 1.1)
High	0.5 (0.3, 0.7)

Kaplan-Meier estimates.

### 3.3 Correlation between JCAD expression and HCC clinical parameters

**Figure 3:**
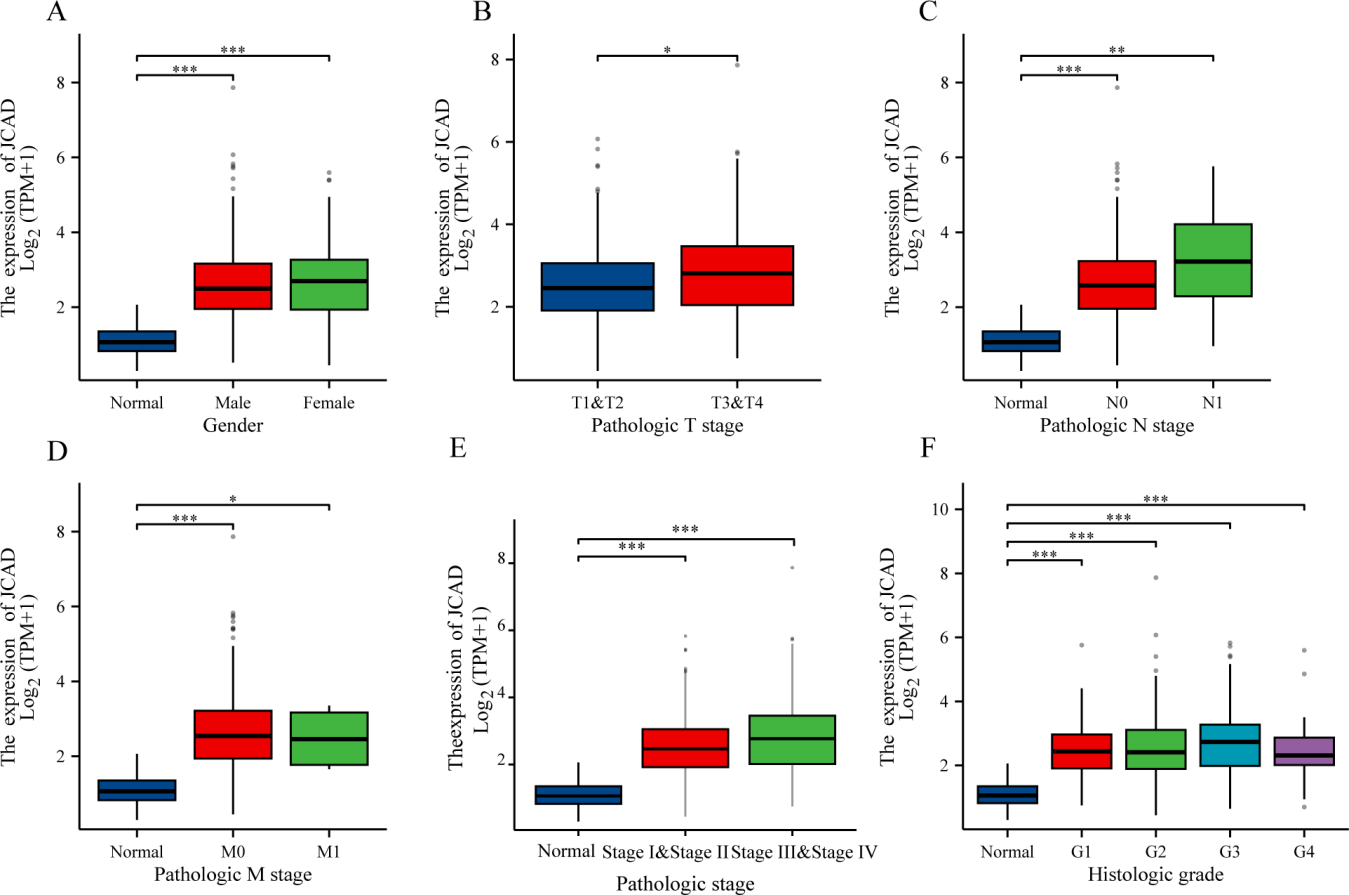
Evaluating JCAD expression in patients with HCC according to different clinical characteristics using the TCGA database. **(A)** Sex. **(B)** T classification. **(C)** N classification. **(D)** M classification. **(E)** Pathologic stage. **(F)** Histologic grade. *p < 0.05; **p < 0.01; ***p < 0.001.

Analysis of clinical data from TCGA, including age, histologic type, grade, and pathologic stage, revealed that JCAD expression was significantly higher in both male and female HCC patients compared to normal controls. Moreover, elevated JCAD levels correlated with advanced TNM staging, with G1-G3 tumors showing higher expression than G0, indicating its association with tumor progression (**Figure 3**).

### 3.4 JCAD correlates with clinical features and outcomes in HCC

**Figure 4:**
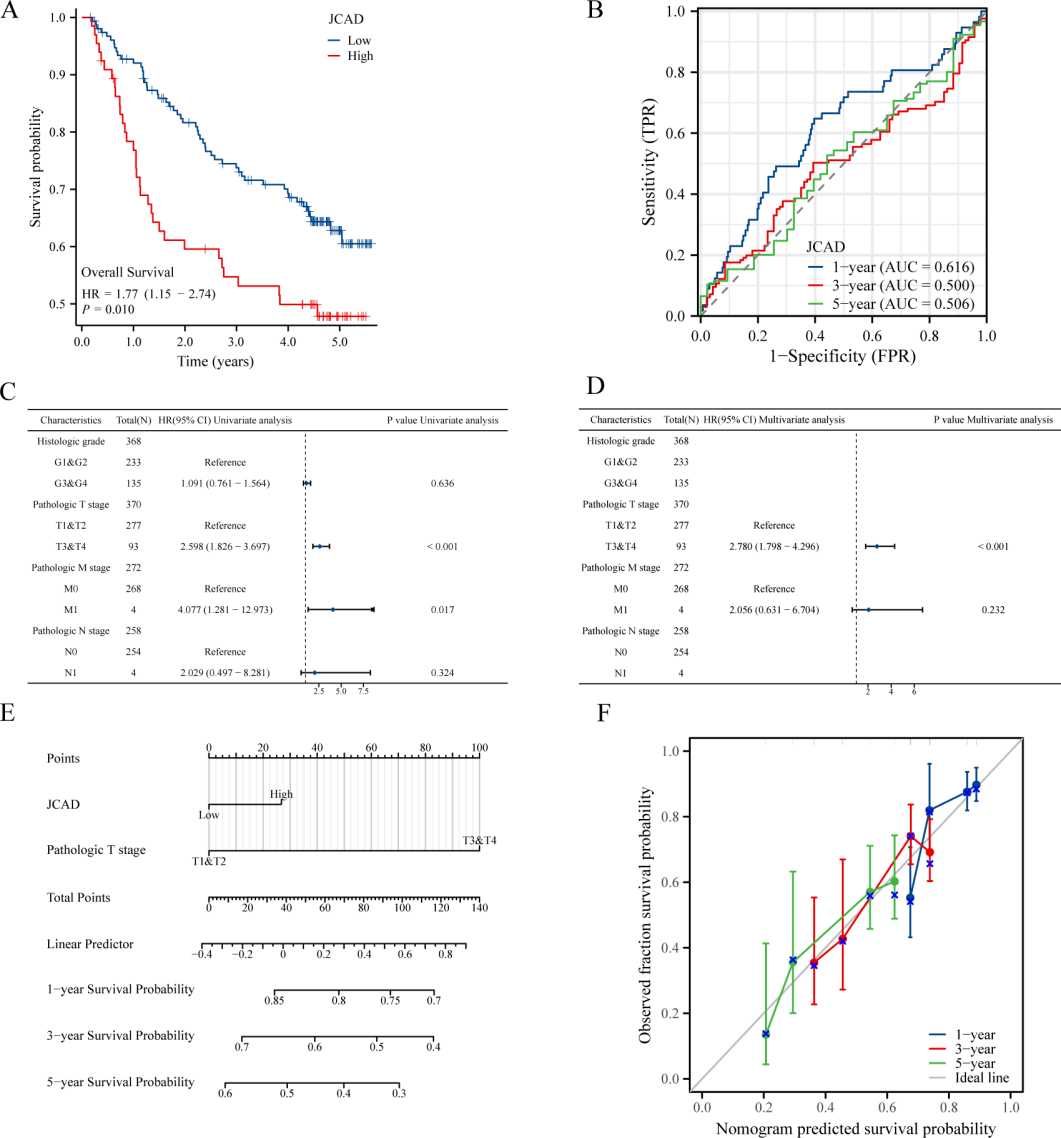
The prognostic value of JCAD and its correlation with clinical features in HCC. **(A)** Further analysis of an external cohort from the Gene Expression Omnibus (GEO) confirmed that elevated JCAD expression was associated with a favorable survival outcome. **(B)** ROC curves of 1-, 3-, and 5-year OS time. **(C, D)** A forest plot of univariate and multivariate Cox regression analyses with OS in HCC from the TCGA database. **(E)** Construction of a prognostic nomogram in HCC. **(F)** Nomogram calibration analysis of HCC prognostic data.

The prognostic significance of JCAD in HCC was further evaluated using Kaplan-Meier survival curves for overall survival (OS). Analysis of an external cohort (GSE14520, n = 488) confirmed that increased JCAD expression was associated with a poorer prognosis for HCC patients. Patients in the JCAD high-expression group exhibited significantly shorter mean OS compared to the low-expression group (**Figure 4A**, p = 0.01). Moreover, the time-dependent ROC curve demonstrated that JCAD expression had prognostic value (**Figure 4B**). Univariate Cox regression analyses further showed that higher JCAD expression was associated with worse prognosis (hazard ratio [HR]: 2.598, 95% confidence interval [CI]: 1.826-3.697), adjusted for pathologic T stage (**Figure 4C**). Similarly, multivariate Cox regression analyses confirmed that JCAD expression was an independent prognostic factor for poorer survival (HR: 2.78, 95% CI: 1.798-4.296), adjusted for pathologic T stage (**Figure 4D**). Based on the multivariate Cox regression analyses, nomogram modes predicting survival were established (**Figure 4E**). Calibration analysis of the nomograms verified the validity of these predictive models (**Figure 4F**). These results confirmed JCAD as an independent prognostic factor for HCC survival.

### 3.5 JCAD-interacting and correlated genes analysis

**Figure 5:**
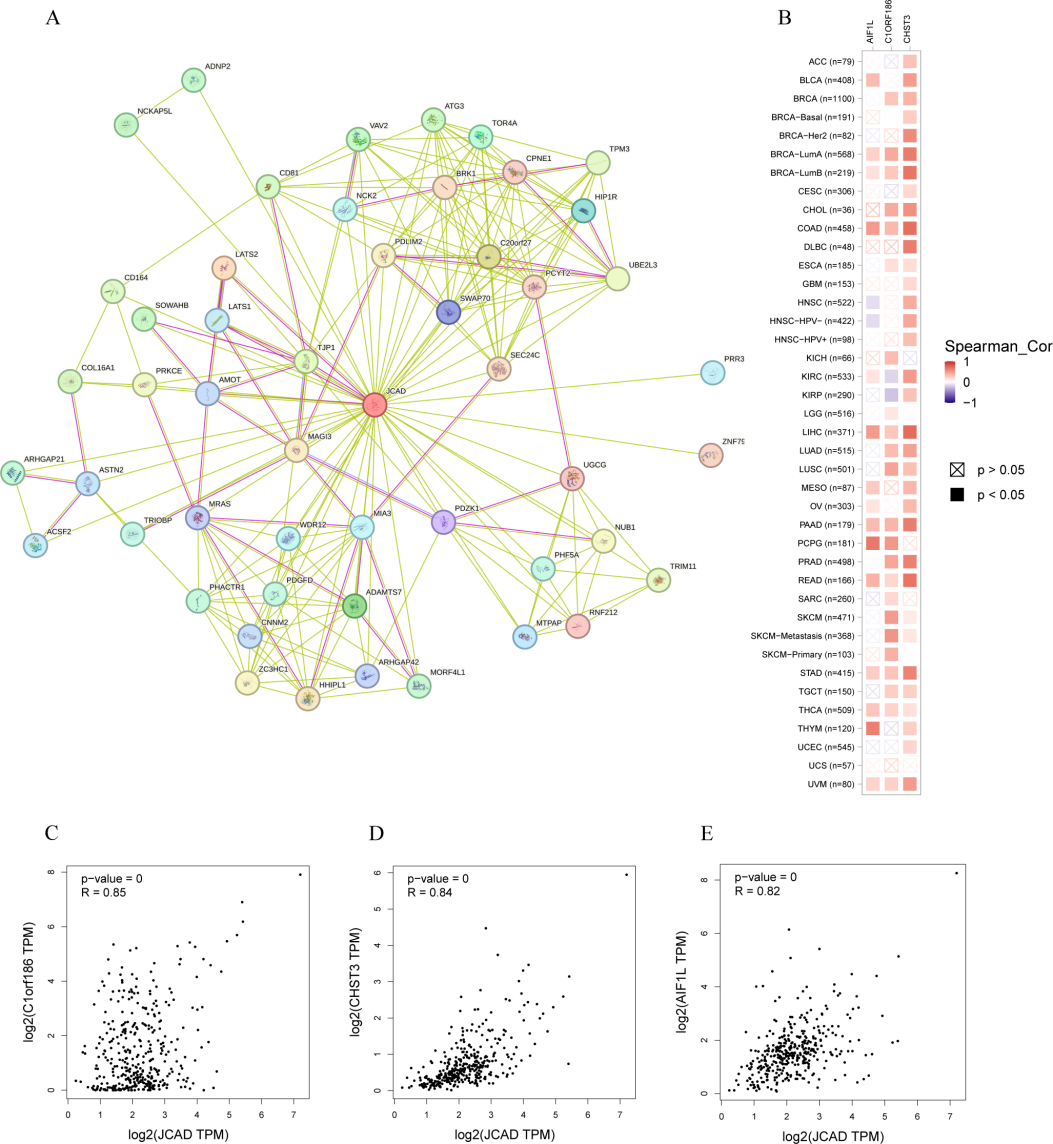
JCAD interaction network and gene co-expression analysis. **(A)** PPI networks of the JCAD-binding proteins were constructed using the STRING tool. **(B)** Heatmap of related genes presented in different tumors. **(C–E)** Correlations between JCAD and the top three JCAD-correlated genes, C1orf186, CHST3, and AIF1L, selected from the top 100 JCAD-related genes in the TCGA project using the GEPIA2 method.

To elucidate the protein-protein interaction (PPI) network of JCAD in HCC, the STRING online tool was employed to construct the PPI network (**Figure 5A**). Using GEPIA2, the top 100 genes co-expressed with JCAD were identified. The heatmap revealed synchronized expression patterns of C1orf186, CHST3, and AIF1L across multiple cancer types (**Figure 5B**). Notably, significant positive correlations were observed between JCAD and C1orf186 (R = 0.85), CHST3 (R = 0.84), and AIF1L (R = 0.82), as demonstrated in scatter plots (**Figures 5C–E**).

**Figure 6:**
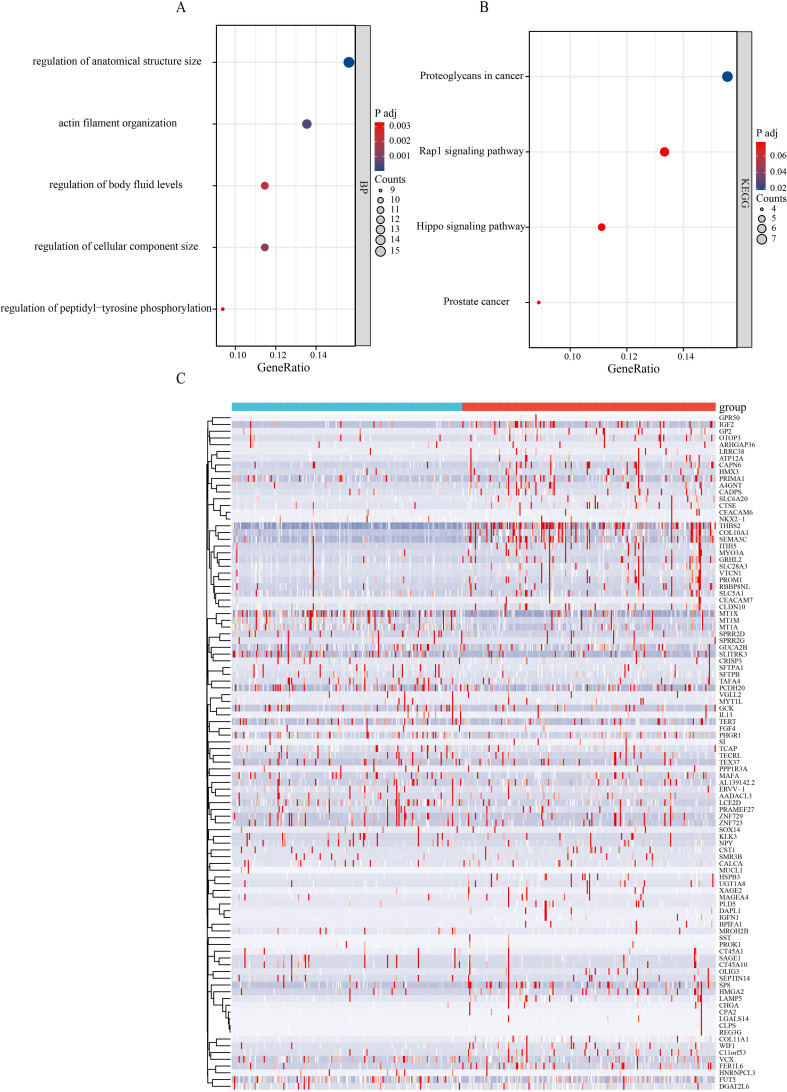
Functional enrichment analysis of JCAD-related genes. **(A, B)** One hudred JCAD-interacting and correlated genes were used for the GO and KEGG enrichment analyses. BP, biological process. **(C)** Heatmap showing the top 50 genes positively and negatively correlated with JCAD in HCC. p < 0.05 were considered statistically significant.

To predict gene function, enrichment analysis of these 100 genes associated with JCAD was conducted (**Figure 6**). Gene Ontology (GO) enrichment analysis revealed that the most enriched GO terms were “regulation of anatomical structure size” and “actin filament organization.” Kyoto Encyclopedia of Genes and Genomes (KEGG) pathway analysis showed that most genes were associated with “proteoglycans in cancer, “ “Rap1 signaling pathway, “ and “Hippo signaling pathway.” GSEA was performed to explore the signaling pathways that are activated by JCAD.

### 3.6 Analysis of correlation between JCAD expression and immune infiltration

**Figure 7:**
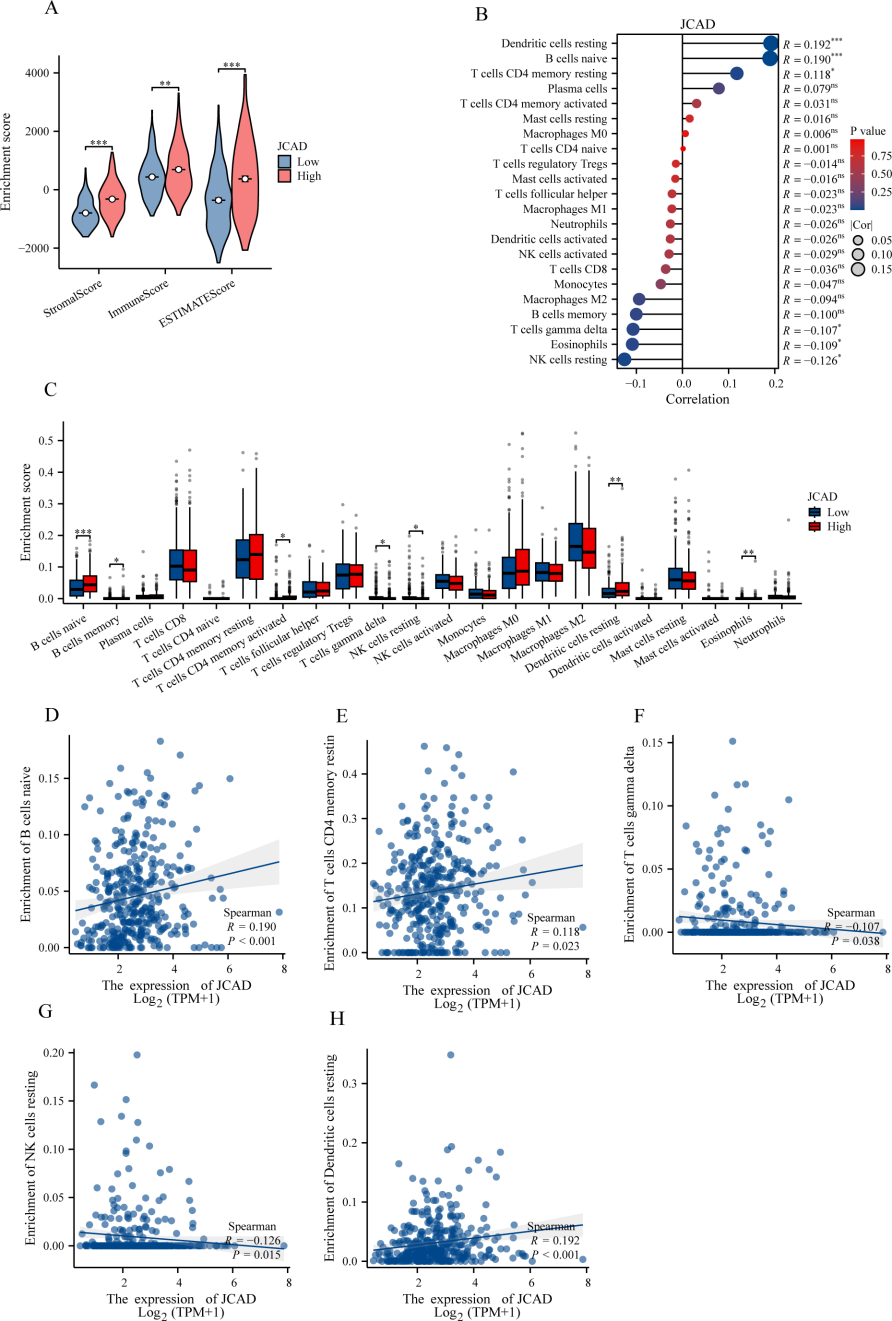
Immune cell infiltration analysis of JCAD. **(A)** Comparison of stroma, immune score and ESTIMATE scores between high and low JCAD expression groups. **(B)** Correlation between JCAD expression and immune cell infiltration. **(C)** Comparison of immune cell infiltration between high and low JCAD expression groups. **(D–H)** Correlation between JCAD expression and various immune cell infiltrations (*p < 0.05; **p < 0.01; ***p < 0.001).

To investigate the impact of JCAD on HCC progression, we first analyzed its role in the tumor microenvironment (TME), which is dominated by tumor cells, immune cells, and stromal cells. Based on data from the TCGA database, the ESTIMATE algorithm was employed to calculate stromal scores, immune scores, and ESTIMATE scores of tumor samples. We then evaluated their correlations with JCAD expression levels. As shown in **Figure 7A**, the stromal scores, immune scores, and ESTIMATE scores in the high-JCAD-expression group were significantly higher than those in the low-expression group (p < 0.01).

Given the increasing role of immunotherapy in cancer, we further examined the association between JCAD and immune-related factors. Using the TCGA database, the CIBERSORT algorithm was applied to analyze the abundance of 22 tumor-infiltrating immune cell types. The results demonstrated that the high-JCAD-expression group exhibited increased infiltration levels of naive B cells and resting dendritic cells, but decreased infiltration of resting NK cells (**Figure 7C**). Further correlation analysis in HCC revealed that JCAD expression was positively correlated with naive B cells (R = 0.19, p < 0.001), resting CD4+ memory T cells (R = 0.118, p = 0.023), and resting dendritic cells (R = 0.192, p < 0.001); it was negatively correlated with gamma delta T cells (R = −0.107, p = 0.038) and resting NK cells (**Figures 7B, D–H**). Moreover, we uncovered notable correlations between the expression of various immune checkpoints and JCAD in patients with HCC (**Supplementary Figures S1A, S1B**), suggesting that JCAD could serve as an indicator of immune checkpoint expression in HCC. We further analyzed the prognostic differences between HCC patients with high and low JCAD expression who are receiving immunotherapy. The results showed that high JCAD expression was significantly associated with poorer prognosis in tumor patients undergoing immunotherapy (**Supplementary Figures S2A, S2B**).

**Figure 8:**
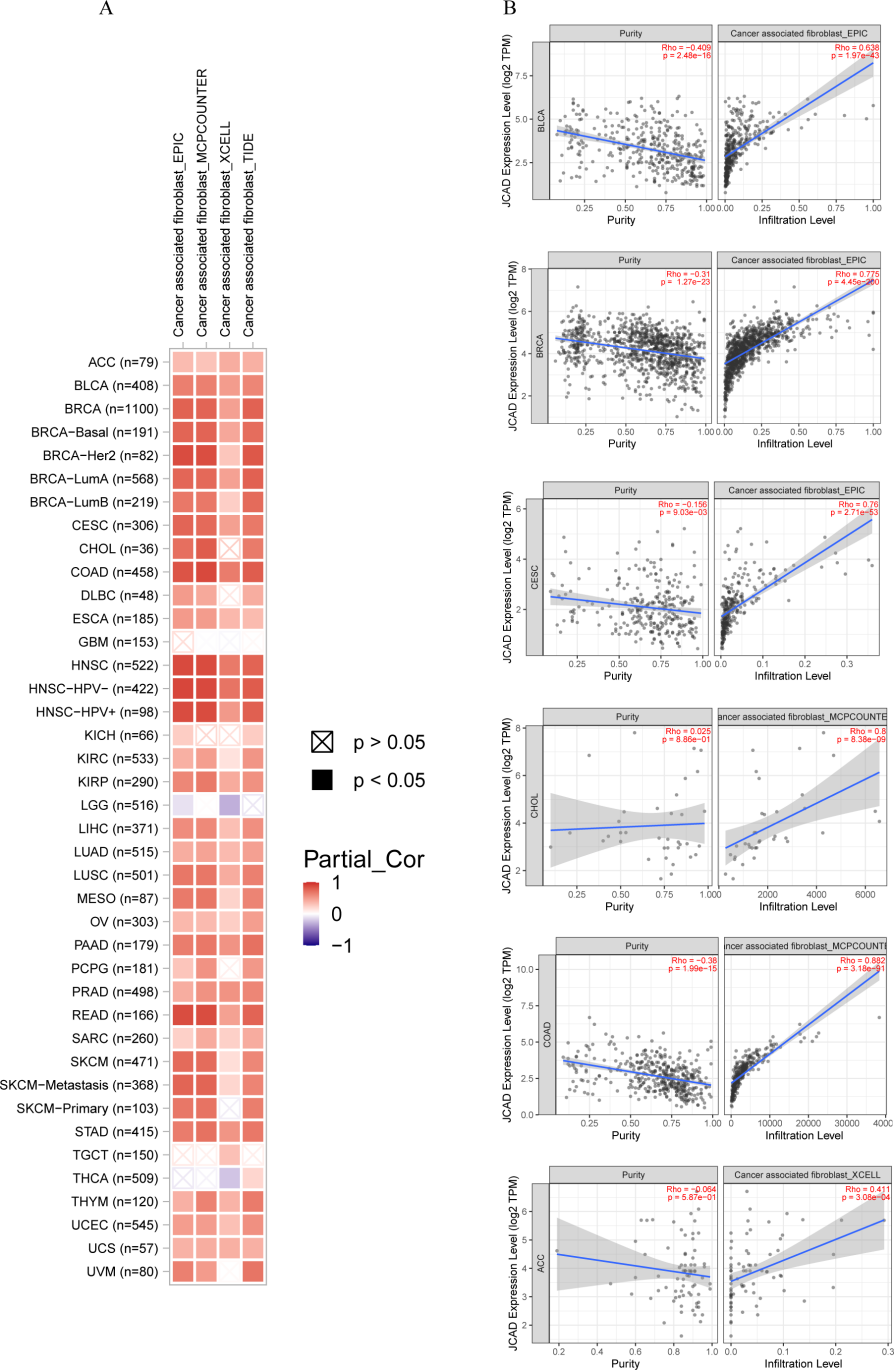
TIMER2-based correlation analysis between JCAD expression and immune infiltration. **(A)** Heatmap of significant positive associations between JCAD expression and CAFs infiltration (BLCA, COAD, HNSC, ESCA, GC). **(B)** Scatter plots of the highest correlation coefficients.

**Figure 9:**
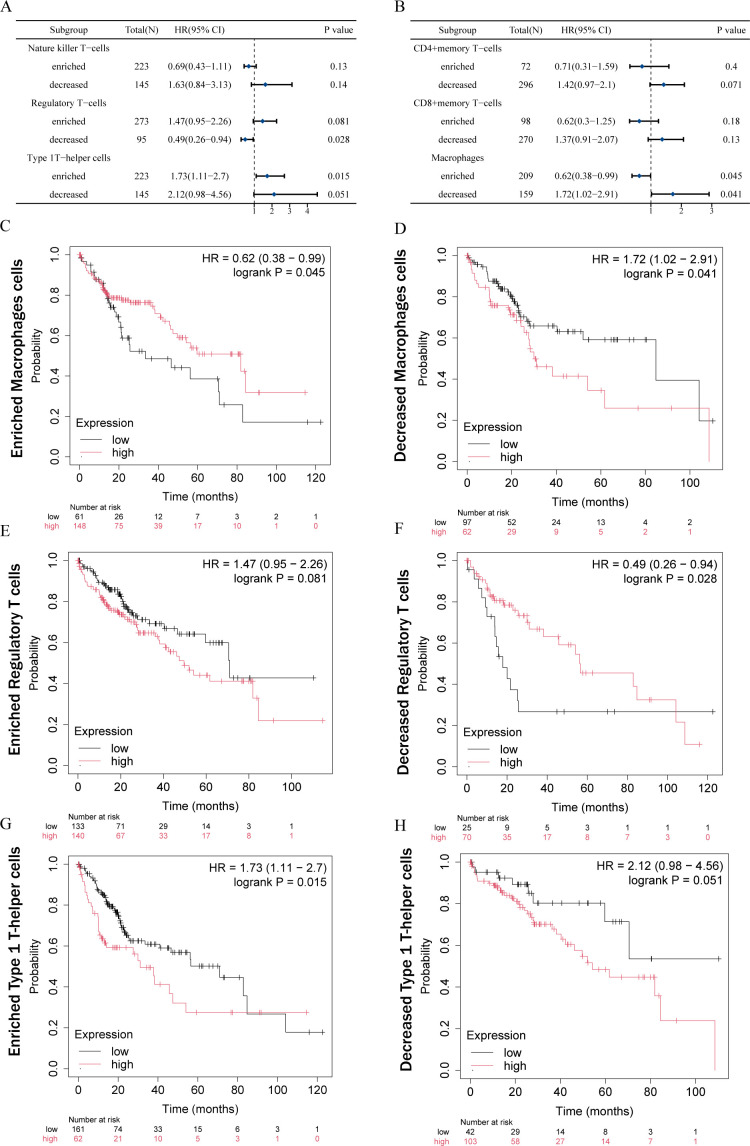
Forest plots summarizing the predictive value of JCAD expression in HCC patients: **(A, B)** show overall analysis, while **(C–H)** represent analyses by different immune cell subsets.

**Figure 10:**
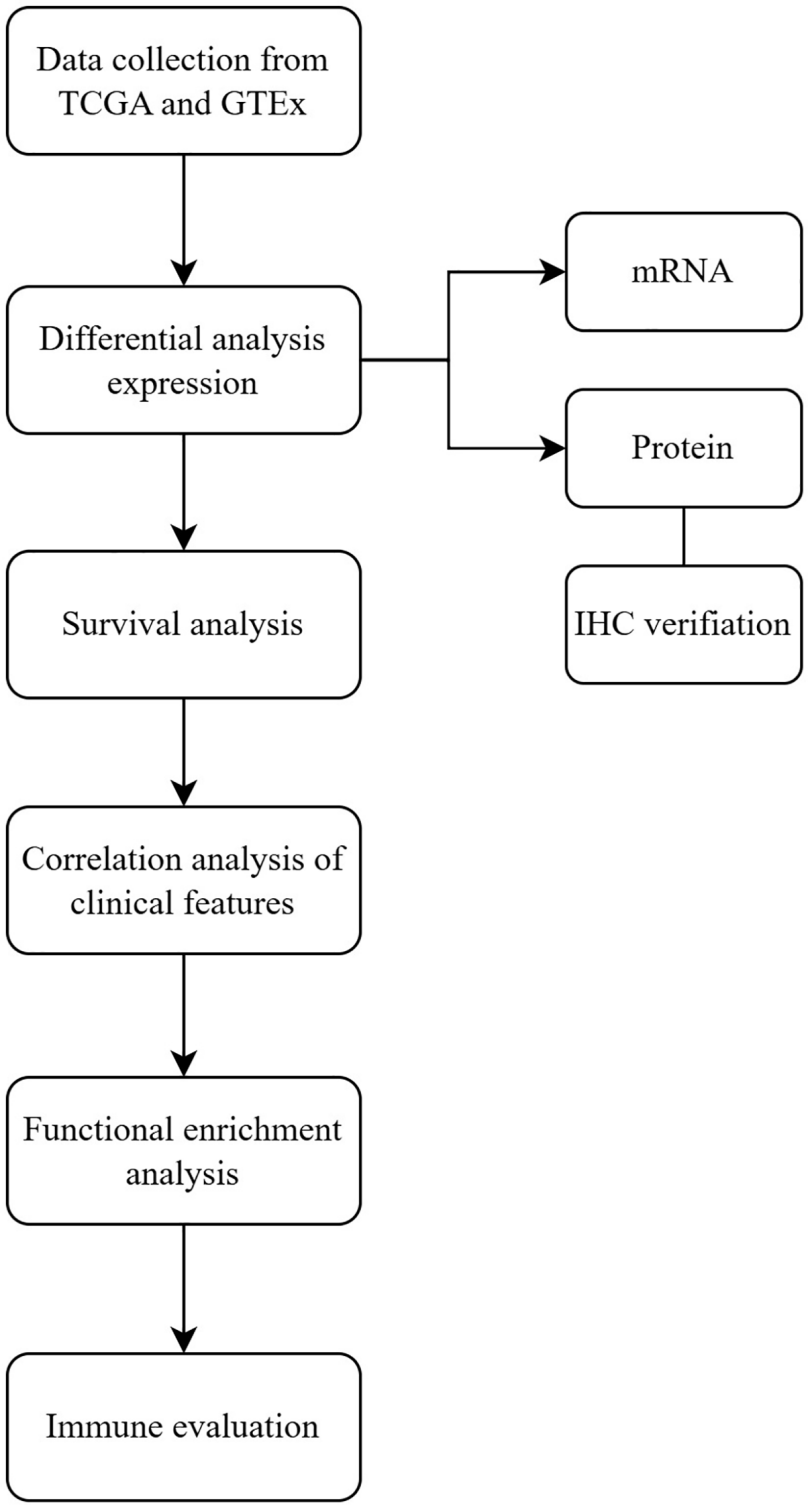
Flow chart.

Furthermore, the TIMER2 algorithm was utilized to explore correlations between JCAD expression and immune cell infiltration across multiple cancer types in the TCGA cohort. This analysis revealed statistically significant positive correlations between JCAD expression and estimates of Cancer-Associated Fibroblasts (CAFs) infiltration in bladder urothelial carcinoma, colon adenocarcinoma, head and neck cancer, esophageal carcinoma, and gastric cancer (GC) (**Figure 8A**). Scatter plots generated using the TIMER2 algorithm, which showed the highest correlation coefficients are detailed in **Figure 8B**. Finally, we investigated whether JCAD expression is associated with HCC patient prognosis through immune cell infiltration. The results indicated that HCC patients with high JCAD expression exhibited increased infiltration of type 1 helper T cells (Th1), reduced infiltration of regulatory T cells (Tregs) and macrophages, and poorer overall survival (**Figure 9**). Ultimately, a flowchart was employed to visually illustrate the investigative pathway of JCAD in HCC (**Figure 10**).

## 4 Discussion

HCC patients face a poor prognosis because of high mortality and recurrence rates. These factors significantly hinder the development of effective therapeutic options (3, 16). The immunosuppressive tumor microenvironment (TME), orchestrated by cells such as tumor-associated macrophages and regulatory T cells, is a key contributor to this poor prognosis and therapy resistance (17). Interestingly, the role of inflammation in HCC is complex. While chronic inflammation promotes carcinogenesis, a stronger inflammatory response within established tumors correlates with increased immune cell infiltration, enhanced cytolytic activity, and improved survival. This highlights the dual nature of inflammatory signals in the TME (18).

Under the evolving paradigm of personalized medicine, the critical importance of predicting disease progression and therapeutic response has become indisputable. Traditional liver biopsy was once considered the gold standard for the diagnosis and monitoring of recurrent HCC. However, due to its invasiveness, sampling variability, and associated complications, it is gradually being replaced by non-invasive methods (19, 20). Clinical guidelines now recommend regular imaging surveillance, with serum alpha-fetoprotein (AFP) testing as an optional adjunct (21). Although studies show reduced HCC-related mortality in screened populations, uncertainties remain about optimal screening protocols. These uncertainties are further complicated by the limited specificity and sensitivity of existing biomarkers. Consequently, identifying robust prognostic biomarkers for HCC remains imperative.

Similar to malignancies such as soft tissue sarcoma (22), recent research highlights inflammatory parameters that may influence tumor aggressiveness and prognosis, including neutrophil-to-lymphocyte ratio (NLR), platelet-to-lymphocyte ratio (PLR), C-reactive protein (CRP), and cytokine profiles (23). However, these biomarkers often exhibit specificity only in niche contexts (e.g., immunotherapy) and have yet to achieve widespread clinical adoption. Multi-gene signatures based on biological processes such as PANoptosis have been shown to stratify HCC patients into distinct immune subtypes with prognostic and immunotherapeutic relevance (24). The most pressing unmet need in HCC management remains the absence of validated, non-invasive biomarkers with clinical utility-markers that combine adequate sensitivity and specificity to reflect HCC progression and prognosis earlier than conventional indicators, thereby optimizing therapeutic decision-making.

JCAD, a cell-junction-associated protein, is overexpressed to varying extents in multiple HCC cell lines. Functional studies using JCAD-knockdown cell models revealed attenuated proliferative capacity and reduced clonogenicity *in vitro (*10, 25). While recent research increasingly links JCAD to HCC pathogenesis, its prognostic significance in HCC remains underexplored. Our clinical validation using immunohistochemistry on 102 paired human HCC and adjacent tissues confirmed JCAD overexpression in 44.1% of tumor samples (**Figure 2A**; **Table 1**). Furthermore, JCAD overexpression was significantly associated with the presence of portal vein tumor thrombus (p = 0.005) and shorter disease-free survival (DFS) (p < 0.001), although it showed no significant correlation with other clinicopathological features such as age, gender, or tumor size (**Table 1**).

To investigate JCAD’s potential role in HCC, we employed Gene Ontology (GO) and Kyoto Encyclopedia of Genes and Genomes (KEGG) analyses. These analyses identified JCAD-associated genes and supported the hypothesis that JCAD expression correlates with immune infiltration in HCC. We confirmed JCAD overexpression in both HCC tissues and cell lines; subsequently, Kaplan-Meier (KM) survival analysis demonstrated its association with poor prognosis. Our KM analysis specifically revealed a significantly reduced DFS for patients with high JCAD expression (log-rank p = 0.0012; **Figure 2B**). The 1-year DFS was 37.8% in the low-expression group, compared to 13.6% in the high-expression group. The 3-year DFS was 2.47% in the low-expression group (**Table 2**). The median DFS was also shorter in the high-expression group (0.5 years) than in the low-expression group (0.8 years) (**Table 3**). A time-dependent ROC analysis yielded an AUC of 0.695 (0.592-0.798) for DFS prediction (**Figure 2C**); collectively, these results indicate that JCAD upregulation is a marker of poor prognosis.

Notably, in the high-JCAD-expression HCC subgroup, we observed increased infiltration of naive B cells and resting dendritic cells, but decreased infiltration of resting NK cells. Further analyses showed that JCAD expression positively correlates with naive B cells, resting CD4+ memory T cells, and resting dendritic cells, but negatively with γδ T cells and resting NK cells. This suggests that JCAD may be associated with an immunosuppressive niche, which aligns with evidence showing that the immunosuppressive TME can be reprogrammed. For example, targeted delivery of CCL3 reshapes macrophage function, enhances antigen presentation, and promotes tertiary lymphoid structure formation, thereby improving T-cell cytotoxicity and response to immune checkpoint blockade (26). Prognostic signatures derived from disulfidptosis-related lncRNAs correlate with immune infiltration and patient survival in HCC (27). Similarly, JCAD expression may also reflect a distinct immunomodulatory profile. Single-cell studies have further classified HCC microenvironments into immune-deserted, B-cell-, T-cell-, and macrophage-enriched subtypes, each with prognostic implications (28). Our observation that high JCAD expression associates with an immunosuppressive cell composition aligns with such heterogeneous immune landscapes. Other studies have identified distinct molecular axes, such as the SNRPB/CCNB1 pathway linked to metabolic reprogramming (29) or PANoptosis-related gene signatures associated with specific immune phenotypes (30). Metabolic reprogramming mediated by splicing factors such as RBM17 can be associated with changes in the immune milieu by promoting M2 macrophage infiltration and suppressing CD8+T cells (31). Whether JCAD influences similar metabolic-immune crosstalk warrants further investigation.

Our data also reveal a significant positive correlation between JCAD expression and multiple immune checkpoints (e.g., PD-1, PD-L1, CTLA-4; **Supplementary Figures S1A, S1B**). In the tumor microenvironment, upregulation of checkpoint molecules often reflects T−cell exhaustion, a state of dysfunctional immune response that promotes immune evasion. While the correlational nature of our findings does not establish a direct regulatory link, we hypothesize that JCAD−high tumors may create a milieu conducive to checkpoint upregulation. Alternatively, JCAD expression could be a surrogate marker for broader immunosuppressive remodeling. Future studies are needed to determine whether JCAD directly influences checkpoint expression or whether this association arises from shared upstream signals.

Critically, JCAD expression correlates with patient survival and shows associations with immune cell infiltration dynamics. This suggests that JCAD could serve as a novel therapeutic target for future combination immunotherapy trials. This is particularly relevant in light of emerging cell therapies; for instance, AFP-targeted TCR T-cell therapy (ADP-A2AFP) has shown manageable safety and preliminary efficacy, yet response rates remain limited, underscoring the need to identify targets like JCAD that could enhance immune cell recruitment and function within the TME (32). We acknowledge that the observed correlations between JCAD expression and immune cell infiltration are hypothesis−generating, and future functional studies—such as co−culture systems or *in vivo* models are required to establish a direct causal relationship. Moreover, previous work has established that JCAD promotes HCC cell proliferation (10). Therefore, the correlations we observed could be secondary to JCAD−driven tumor growth rather than a direct immunomodulatory effect. Highly proliferative tumors often exhibit altered tumor purity, stromal composition, and hypoxia, which may indirectly influence immune cell recruitment. Our current bioinformatic analysis cannot distinguish between direct and indirect effects. Thus, we explicitly note that the relationship between JCAD and immune infiltration may be confounded by tumor growth dynamics. Future studies employing co−culture systems (e.g., JCAD−manipulated HCC cells with immune cells) or conditional JCAD knockout *in vivo* are needed to dissect causality.

## 5 Conclusion

In conclusion, we integrate bioinformatic analysis with clinical validation to propose JCAD as a dual-function prognostic biomarker and potential therapeutic target in HCC. Immunohistochemistry confirmed that JCAD is overexpressed in 44.1% of tumors. This overexpression was clinically associated with portal vein tumor thrombus and significantly reduced disease-free survival. Specifically, the median DFS was 0.5 years in high-expression patients versus 0.8 years in low-expression patients (log-rank p = 0.0012). Mechanistically, JCAD expression correlates positively with specific immune cell infiltration, suggesting its prognostic role may be correlated with features of the tumor immune microenvironment. These findings collectively underscore the clinical relevance of JCAD and warrant further functional studies to elucidate its precise oncogenic mechanisms and therapeutic applicability.

## Data Availability

The original contributions presented in the study are included in the article/**Supplementary Material**. Further inquiries can be directed to the corresponding authors.
