# Supplementary material for: Study of JCAD for prognosis and immune infiltration in hepatocellular carcinoma
Source: Front Immunol. 2026 May 28;17:1831629. doi: 10.3389/fimmu.2026.1831629 (PMC13254190; doi:10.3389/fimmu.2026.1831629)
Supplement: Supplementary file 1 [file DataSheet1.docx]

**
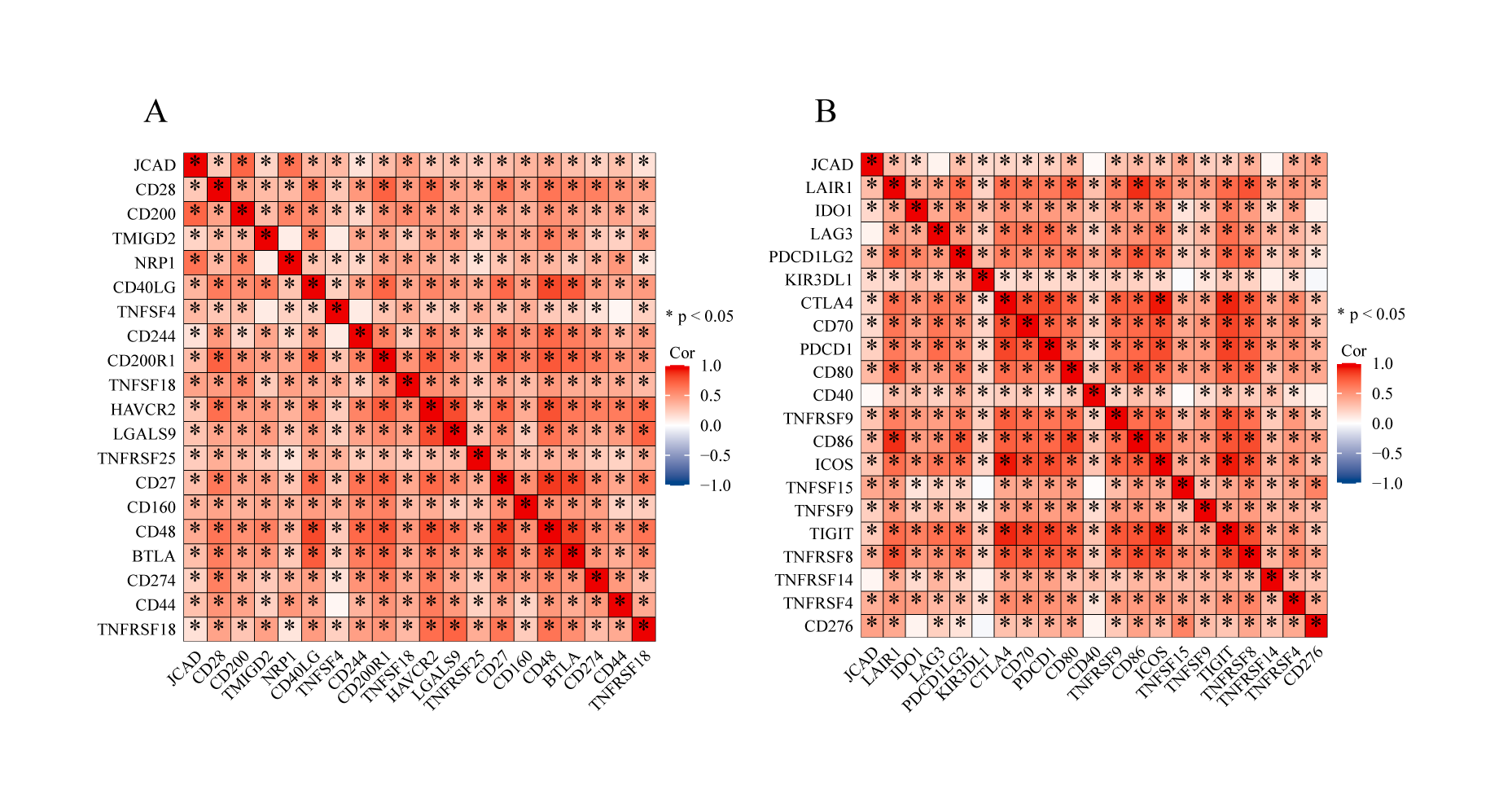
**

**FIGURE S1 (A, B)** Relationship between JCAD expression and immune checkpoint genes (*p < 0.05; **p < 0.01; ***p < 0.001).

**
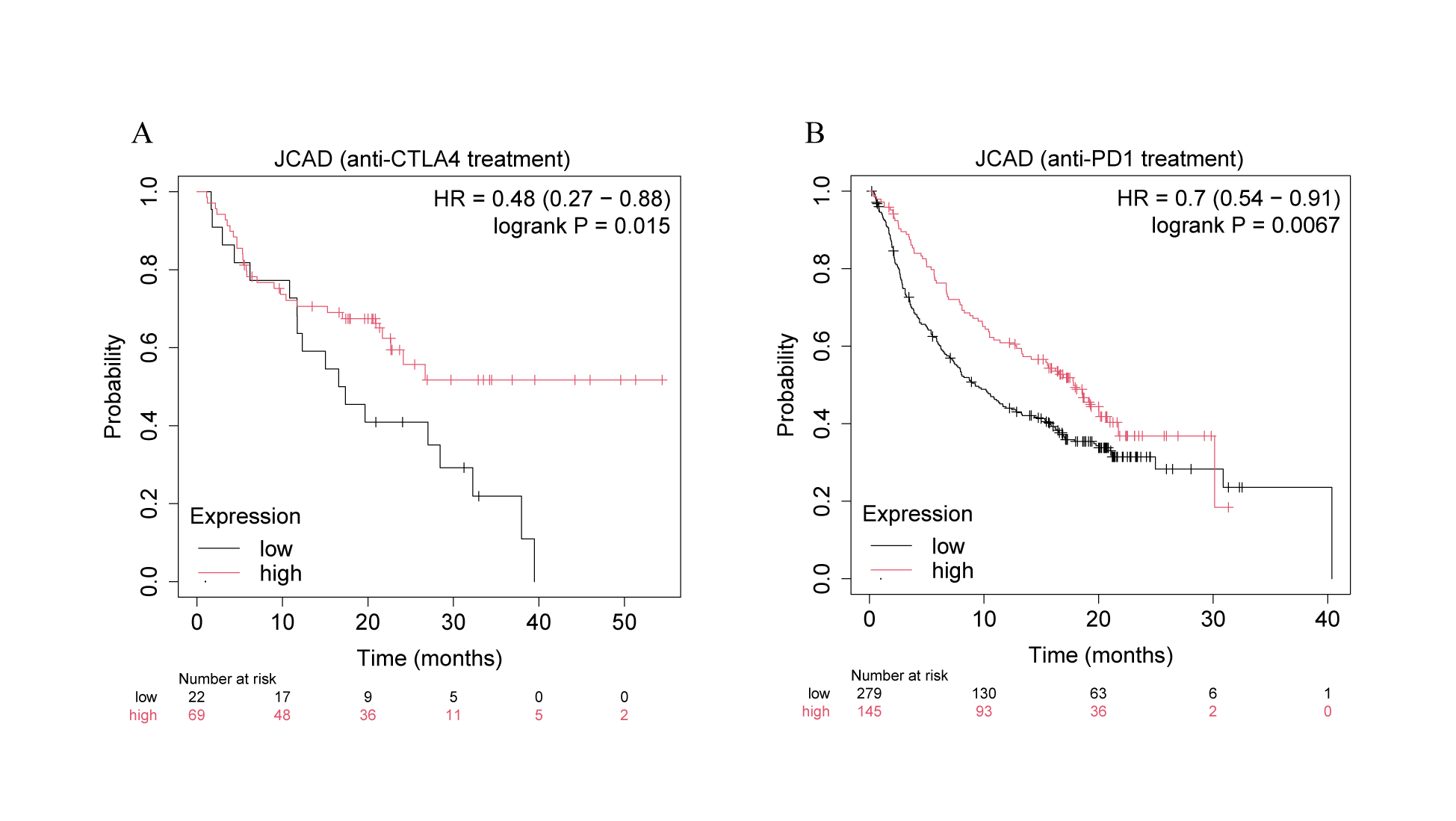
**

**FIGURE S2 (A, B)** Comparison of prognosis for IPS-CTLA4 blocker and IPS-PD1 blocker between JCAD high and low expression groups.
